# Supplementary material for: Tead1a Initiates Transcriptional Priming Through the TEAD1a/YAP‐Notch1‐Spi1/Cebpα Axis to Promote Neutrophil Fate
Source: Adv Sci (Weinh). 2025 Aug 28;12(41):e05441. doi: 10.1002/advs.202505441 (PMC12591127; doi:10.1002/advs.202505441)
Supplement: Supplementary file 4 — Supplementary Dataset [file ADVS-12-e05441-s004.zip › 3-3.zip!/3-3/02-3.cluster/cluster8/Enrich/ego_all.pdf]

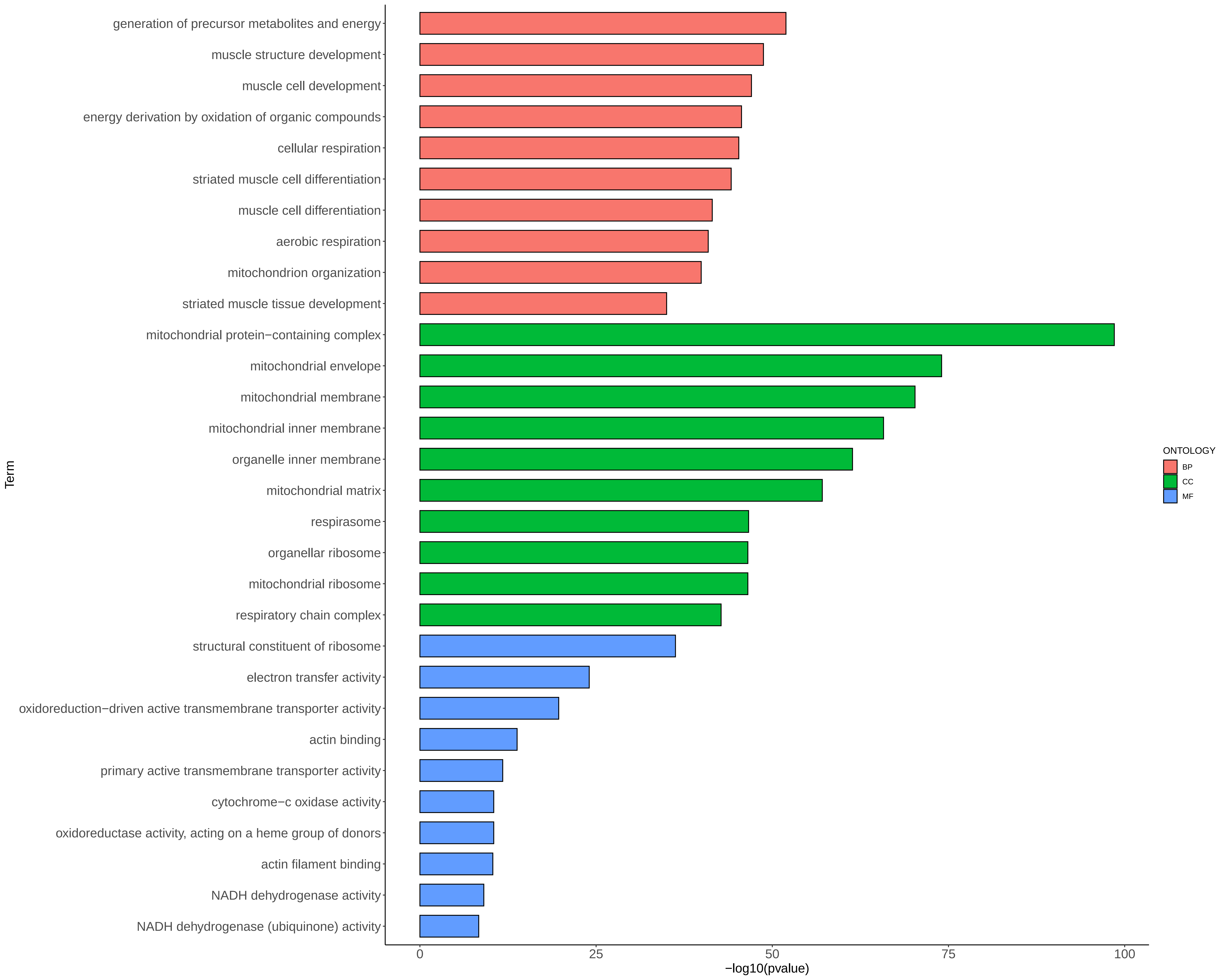
generation of precursor metabolites and energy
muscle structure development
muscle cell development
energy derivation by oxidation of organic compounds
cellular respiration
striated muscle cell differentiation
muscle cell differentiation
aerobic respiration
mitochondrion organization
striated muscle tissue development
mitochondrial protein−containing complex
mitochondrial envelope
mitochondrial membrane
mitochondrial inner membrane
organelle inner membrane
mitochondrial matrix
respirasome
organellar ribosome
mitochondrial ribosome
respiratory chain complex
structural constituent of ribosome
electron transfer activity
oxidoreduction−driven active transmembrane transporter activity
actin binding
primary active transmembrane transporter activity
cytochrome−c oxidase activity
oxidoreductase activity, acting on a heme group of donors
actin filament binding
NADH dehydrogenase activity
NADH dehydrogenase (ubiquinone) activity
Term
0
25
50
75
100
−log10(pvalue)
ONTOLOGY
BP
CC
MF
